# Synergetic Effects during Co-Pyrolysis of Sheep Manure and Recycled Polyethylene Terephthalate

**DOI:** 10.3390/polym13142363

**Published:** 2021-07-19

**Authors:** Zuhal Akyürek

**Affiliations:** Department of Energy Systems Engineering, Faculty of Engineering and Architecture, Burdur Mehmet Akif Ersoy University, Burdur 15030, Turkey; drzuhalakyurek@gmail.com

**Keywords:** co-pyrolysis, synergy, kinetics, plastic waste, animal manure

## Abstract

Continuous growth in energy demand and plastic waste production are two global emerging issues that require development of clean technologies for energy recovery and solid waste disposal. Co-pyrolysis is an effective thermochemical route for upgrading waste materials to produce energy and value added products. In this study, co-pyrolysis of sheep manure (SM) and recycled polyethylene terephthalate (PET) was studied for the first time in a thermogravimetric analyzer (TGA) in the temperature range of 25–1000 °C with heating rates of 10–30–50 °C min^−1^ under a nitrogen atmosphere. The synergetic effects of co-pyrolysis of two different waste feedstock were investigated. The kinetic parameters are determined using the Flynn–Wall–Ozawa (FWO) model. The results revealed that the mean values of apparent activation energy for the decomposition of sheep manure into a recycled polyethylene terephthalate blend are determined to be 86.27, 241.53, and 234.51 kJ/mol, respectively. The results of the kinetic study on co-pyrolysis of sheep manure with plastics suggested that co-pyrolysis is a viable technique to produce green energy.

## 1. Introduction

Depletion of fossil fuel reserves (petroleum, coal, natural gas) together with the environmental concerns of fossil fuel combustion have diverted attention towards renewable energy sources worldwide. According to the International Energy Agency (IEA), Global CO_2_ emissions from fuel combustion reached 33.5 GtCO_2_ carbon dioxide emissions, and 40% of the emissions stem from electricity generation, driven by factors such as electricity output, generation efficiency, and carbon intensity of fossil fuel generation [1]. Biomass energy is one of the emerging alternatives for reduction of CO_2_ emissions and diversification of energy sources. Biomass can contribute to sustainable development while reducing climate change impacts on industry [2]. Biomass is the fourth largest energy system after coal, oil and gas with a share of 14% in global energy consumption. Electricity generation from bioenergy is predicted to show an annual increase of 6% through to 2030 in the Sustainable Development Scenario (SDS) [3].

Solid waste generation increases gradually due to population growth, developments in industry and enhanced living standards. Carbon neutral energy sources such as biomass can be utilized to address the issues of energy production and waste management [4]. Biomass is an abundant source that can be converted into energy. Organic materials such as agricultural crops, organic wastes, forest residues and livestock manure can be used as biomass feedstock. Livestock manure is a challenging biomass that needs to be carefully managed in order to minimize the greenhouse gas emissions (CH_4_, N_2_O), adverse health effects and pollution of aquifers and surface waters [5,6].

Plastic waste is one of the fastest-growing environmental pollutant materials. Plastic production has increased in the last decades, due to the applications of several industries such as packaging, construction, buildings, electronics, textiles, machinery etc. Degradation of plastics changes from weeks to several years. Global plastics production almost reached 370 million tons, and in Europe plastics production almost reached 58 million tons [7]. The continuous rise in plastics consumption has led to adverse effects on the environment [8,9]. Plastic wastes can be managed through recycling or energy recovery methods [10]. Recycling is a possible way of plastic waste disposal. Nevertheless, recycling processes are generally costly, energy intensive, and the quality of the product is low [11]. The plastics have high calorific value because they are produced from petrochemical sources. Plastic waste generally ends up in landfills. Lost energy in landfills is estimated to be 2.8 quads of energy equivalence [4]. Hence, energy production from plastic waste has gained interest, to minimize the waste and energy loss.

Thermochemical conversion methods can be used for waste treatment in order to eliminate plastic waste. Combustion or incineration of plastic materials may generate harmful emissions to the atmosphere. Pyrolysis, on the other hand, is one of the viable thermal treatment routes for waste minimization and energy recovery from solid waste [12,13]. Pyrolysis is a precursor for combustion and gasification processes [14]. During pyrolysis, long chain polymers degrade into smaller molecules in oxygen free environments [15]. The major products that are produced during pyrolysis are bio-oil, synthetic gas, and biochar products. Depending on the heating rate and residence time, pyrolysis can be classified into three categories: slow, fast and flash pyrolysis [16]. Slow pyrolysis is generally applied for biochar production. Thermal decomposition of biomass takes place under low heating rates and sufficient residence time for re-polymerization reactions in order to increase the yield of solid product termed as biochar. Biochar is to be used in various applications such as soil amendment, energy production and control of pollutants [17]. Fast and flash pyrolysis generally produces higher amounts of bio-oil [18]. Biomass pyrolysis is a complex process due to varying reaction mechanisms and reaction rates during decomposition of the different biomass components [19]. Pyrolysis efficiency also depends on the operational conditions and reactor design.

Co-pyrolysis is considered to be an easy and safe process for producing high quality fuels [20]. Biomass/plastics co-pyrolysis is as an effective upgrading method that will not only increase the bio-oil yield but also reduce activation energy compared to individual pyrolysis of raw materials [4]. In the study of Zhang et al. [21], thermal decomposition behavior and kinetics of sawdust and plastic waste co-pyrolysis was investigated by using a thermogravimetric analyzer (TGA), and synergistic interactions were detected during co-pyrolysis. Aboulkas et al. [22] carried out olive residue/plastic waste (high and low density polyethylene, polypropylene and polystyrene) co-pyrolysis experiments in TGA. The results indicated significant synergy interactions at the high temperature region. Alam et al. [23] studied co-pyrolysis of bamboo sawdust and linear low-density polyethylene in TGA. Synergistic interactions were more obvious with the blend 25 wt% bamboo sawdust. Uzoejinwa et al. [24] reviewed the benefits of the co-pyrolysis process, product yields, mechanisms of biomass with plastics, and synergetic effects during co-pyrolysis. They stated that co-pyrolysis could serve as a solid waste management method for reducing waste inventory and reducing the dependency on fossil fuels.

Plastics and animal manure can cause detrimental effects on the environment and threaten public health. Their utilization in co-pyrolysis processes provides the reduction of pollutants, on one hand, and recovers green energy, on the other. Kinetics of conversion is essential to understanding the pyrolysis of biomass and plastic materials. In this study, co-pyrolysis characteristics of sheep manure and polyethylene terephthalate (PET) were investigated and identified from thermogravimetric analysis coupled with kinetic study.

## 2. Materials and Methods

### 2.1. Raw Materials

**Figure 1:**
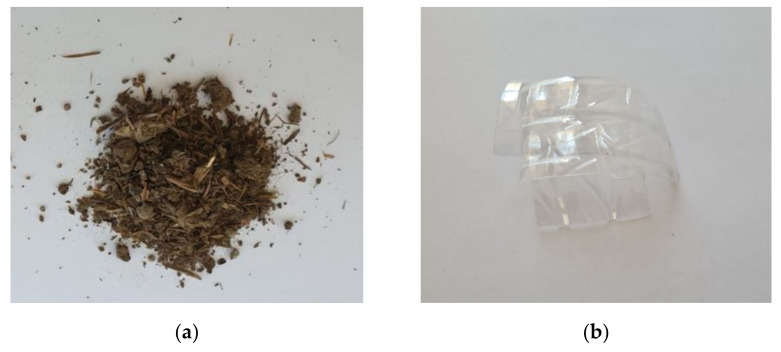
Photograph of raw materials (**a**) SM, (**b**) PET.

Livestock farming has a high contribution to the economy in Turkey. The availability of animal manure signifies its potential for energy production. In this study, sheep manure (SM) (Figure 1a) and Polyethylene terephthalate (PET) (Figure 1b) were used as raw materials. Manure was obtained from the Koç Family Farm in Ağlasun, Burdur, Turkey. The sample was dried in oven at 80 °C overnight, and then a piece was cut out which was 0.5–1 mm size. Polyethylene terephthalate (PET) samples were reduced into the similar size range with the sheep manure (SM).

In Table 1, the proximate and elemental analyses of the samples are shown. Moisture, volatile matter, and ash contents of the feedstock were determined by ASTM D3173, ASTM D 3175, ASTM D 3174, respectively. The major elements (C, H, N, S) were detected by the LECO CHNS-932 elemental analyzer, and the content of O was calculated by the difference. The calorific value of the samples was determined in the LECO AC-350 Bomb Calorimeter.

### 2.2. Thermogravimetric Analysis

Thermogravimetric (TG) and derivative thermogravimetric (DTG) analysis experiments of the sheep manure and recycled polyethylene terephthalate were carried out using a TG analyzer (Seiko SII TG/DTA 7200) under a nitrogen flow of 100 mL/min, heated from room temperature up to 900–1000 °C. The experiments were carried out at three different heating rates (10, 30 and 50 °C/min). The sample weight was kept at about 10 mg.

The evolution with temperature of weight loss (TG) and the weight loss rate (DTG) were obtained for pyrolysis. The weight loss rate was calculated as [25]:

(1) $$\left(\frac{dW}{dt}\right)=-\frac{1}{w_{0}}\;\left(\frac{dm}{dt}\right)$$

where *W*_0_ is the initial sample mass.

The synergistic effects during co-pyrolysis of (50:50 wt%) SM and PET blend was determined by calculating the difference of weight loss (Δ*W*) on the basis of each material during pyrolysis [26];

(2) $$\Delta W=W_{Blend}-\left(\frac{W_{1}+W_{2}}{2}\right)$$

### 2.3. Kinetic Analysis

In order to estimate the kinetic parameters of pyrolysis, the isoconversional methods are commonly applied. In this study, the Flynn–Wall–Ozawa (FWO) method was used to estimate the apparent activation energy of SM, PET and their blend (50:50 wt%). Pyrolysis kinetics of biomass can be expressed according to the Arrhenius relation, *k*(*T*) as;

(3) $$k\left(T\right)=Aexp\left(\frac{-E}{RT}\right)$$

where *T*(*K*) is the absolute temperature, *k*(*T*) is the reactivity (the rate constant) depending on the temperature, *A* (s − 1) is the pre-exponential factor, *E* (J/mol) is the activation energy and R is the universal gas constant (8.314 KJ/mol K).

The kinetics of heterogeneous solid-state thermal degradation is dominated by the fundamental equation [22];

(4) $$\frac{d\alpha }{dt}=k\left(T\right)f\left(\alpha \right)$$

(5) $$\frac{d\alpha }{dt}=Aexp\left(\frac{-E_{A}}{RT}\right)f\left(\alpha \right)$$

where *t* is the time, and *f*(*α*) is the reaction function depending on the conversion rate *α* in relation to the reaction model, at the conversion degree *α*.

The conversion for pyrolysis is described as;

(6) $$\alpha =\frac{W_{0}-W_{t}}{W_{0}-W_{f}}$$

where *W*_0_ and *W_f_* are the initial and final weight of the sample, respectively. *W_t_* is the weight of the sample at temperature *T*.

Heating rate *β* (K/s) is defined as;

(7) $$\beta =\frac{dT}{dt}$$

Equation (2) can be transformed into;

(8) $$\frac{d\alpha }{dT}=\frac{A}{\beta }exp\left(\frac{-E_{A}}{RT}\right)f$$

The integrated form of *f*(*α*) is generally expressed as;

(9) $$G\left(\alpha \right)=\overset{\alpha }{\underset{0}{\int }}\frac{d\left(\alpha \right)}{f\left(\alpha \right)}=\frac{A}{\beta }\overset{T}{\underset{T_{0}}{\int }}exp\left(\frac{-E}{RT}\right)dT$$

#### 2.3.1. The Flynn–Wall–Ozawa (FWO) Method

Flynn–Wall–Ozawa (FWO) is an integration method, which provides a linear correlation for a given value of conversion at different heating rates [27,28];

(10) $$In\beta =In\frac{AE_{a}}{Rg\left(\alpha \right)}-5.331-1.052\frac{E_{a}}{RT}$$

The apparent activation energy can be calculated from the plot of *Inβ* vs. 1/*T* for a given value of conversion, where the slope is equal to −1.052 *E_a_*/*R*.

#### 2.3.2. Thermodynamic Parameters

The pre-exponential factors (*A*) and other thermodynamic parameters such as Enthalpy (Δ*H*), Gibbs free energy (Δ*G*), and entropy (Δ*S*) were calculated by Equations (11)–(14)

(11) $$A=\beta .E_{a}.\exp\left(\frac{E_{a}}{R.T_{max}}\right).\frac{1}{R.T_{max}^{2}}$$

(12) $$\Delta H=E_{a}-RT$$

(13) $$\Delta G=E_{a}+R.T_{max}.In\left(\frac{K_{b}.T_{max}}{h.A}\right)$$

(14) $$\Delta S=\frac{\Delta H-\Delta G}{T_{max}}$$

where *T_max_* is the peak temperature, KB is the Boltzmann constant (1.381 × 10^−23^ J/K) and h is the Plank constant (6.626 × 10^−34^ J.s).

## 3. Results and Discussion

### 3.1. Pyrolysis and Co-Pyrolysis of SM and PET

**Figure 2:**
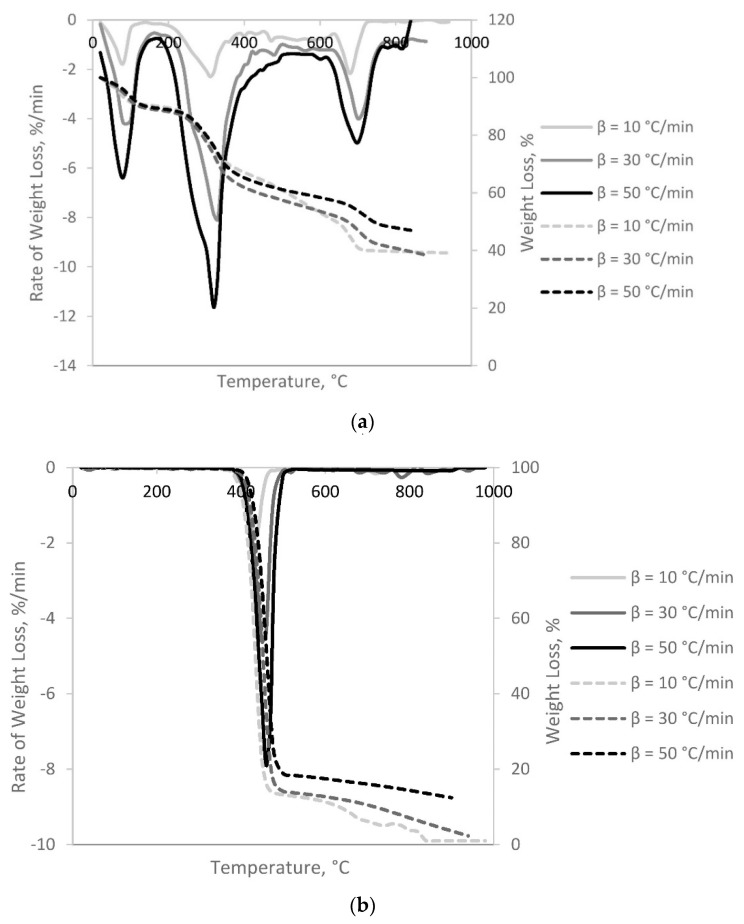
TG and DTG Graphs of (**a**) SM and (**b**) PET at different heating rates.

Thermogravimetric analysis is a useful method in order to explain the thermal decomposition of the fuel and reaction mechanisms, which occurred during pyrolysis. The *TG* curves shows the weight change with respect to temperature change during thermal degradation, and the *DTG* curves show the corresponding rates of mass loss of the *TG* curves. In Figure 2, pyrolysis behavior of the sheep manure and polyethylene terephthalate were presented with the mass loss (*TG*) and derivative mass loss (*DTG*) curves under different heating conditions.

Pyrolysis behavior and characteristic temperatures such as initial decomposition temperature (*T_in_*), peak temperature (*T_max_*), and final temperature (*T_f_*) were obtained from TGA-DTG profiles, as listed in Table 2.

Figure 2 showed that thermal decomposition of SM and PET differ from each other. Sheep manure (SM) was mainly composed by hemicellulose, cellulose, lignin, and some other organic materials. In contrast, PET was a long linear polymer with a high degree of crystallinity and low branching [12]. SM degraded at a lower temperature and in a broader decomposition range compared to PET, which was consistent with previous studies [29,30]. The DTG curve for SM can be divided into three main stages. In the first stage (up to 200 °C), there occurred evaporation of free moisture, primary decomposition of unstable biopolymers, followed by devolatilization due to secondary reactions such as cracking and repolymerization [31]. In the second stage (200–450 °C) the main weight loss was observed, where the active pyrolysis occurred by devolatilization, cellulose, hemicellulose and partial lignin degradation, which are the major components in the waste material. The third stage (600–800 °C) corresponded to the continuous devolatilization with lignin degradation and char formation. Similar results were reported for biomass pyrolysis in previous studies [21,26]. Single peaks observed in the temperature range of 400–500 °C in DTG of PET suggested the overall single step degradation. Degradation characteristics of PET were found to be similar for polymer degradations such as LDPE and HDPE [32,33]. SM showed typical biomass characteristics, and decomposed at lower temperatures than PET. This was attributed to the structure of PET, which is less complicated compared to that of biomass. The heating rate is an important factor during pyrolysis. As can be seen from Table 2, the maximum temperature shifted towards higher values by increasing the heating rate without altering thermal decomposition [23,34,35].

Synergetic effects of the manure and PET co-pyrolysis was investigated by comparing the theoretical and experimental thermogravimetric analysis results. The theoretical values of the blends was calculated by the weighted-average sum of the individual sample’s TGA experiment values. PET blending with SM increased the rate of volatile evolution during manure decomposition, and lowered the peak corresponding to pyrolysis compared to the weighted DTG.

**Figure 3:**
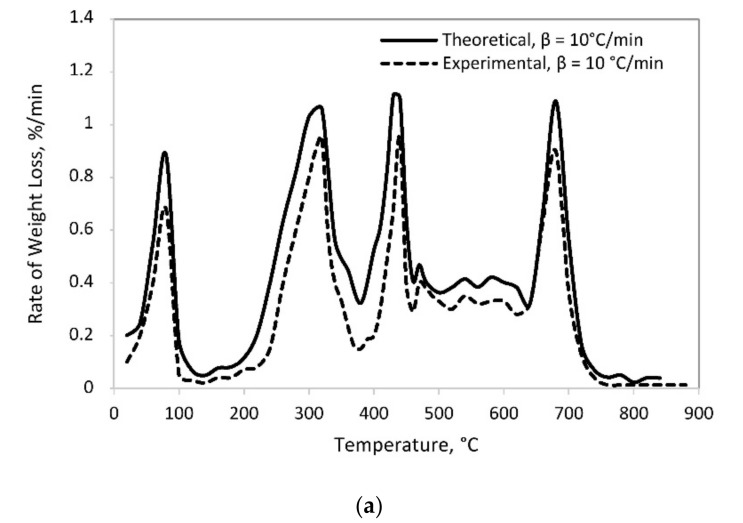

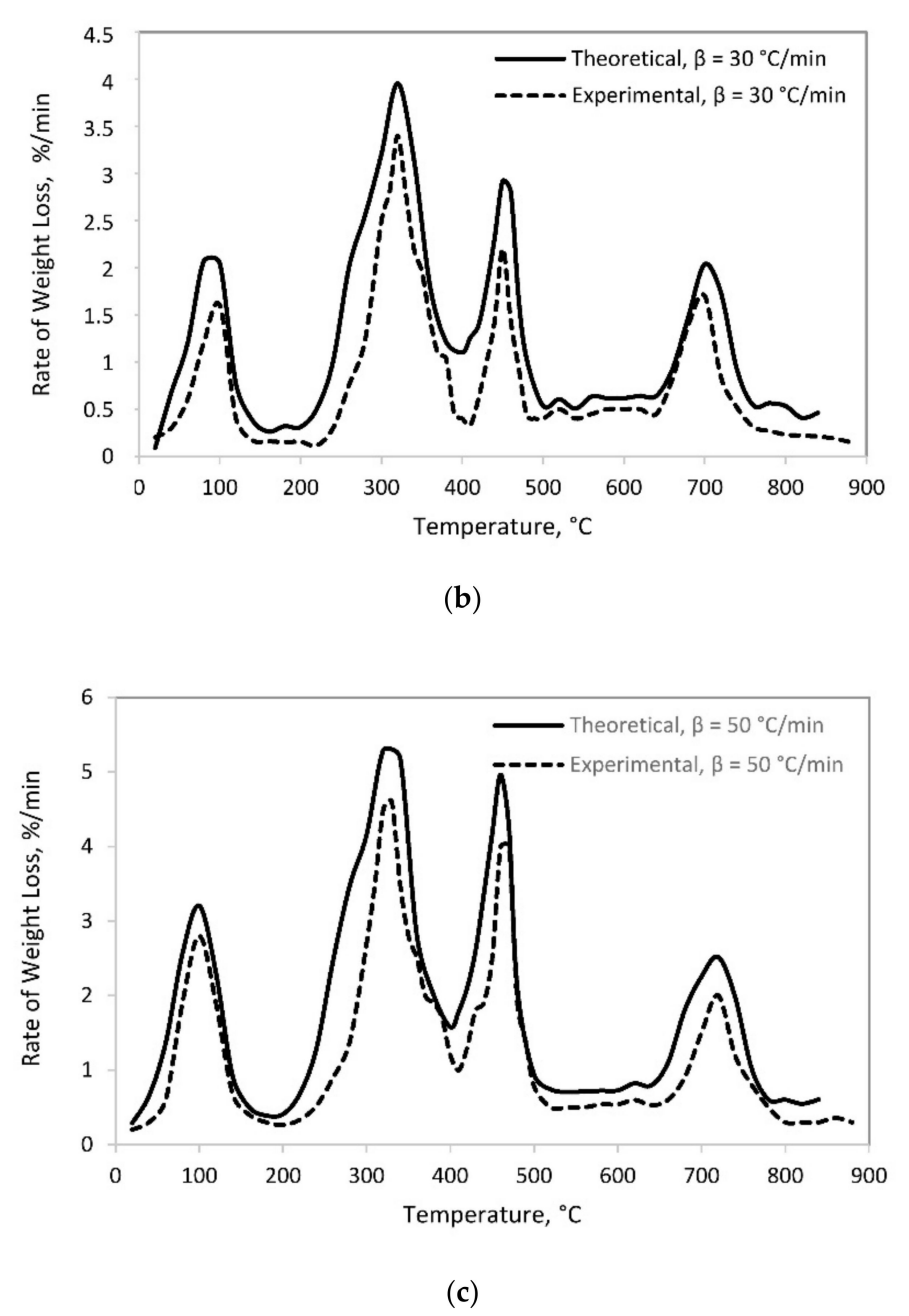
Comparison of theoretical and experimental data of SM/PET blend (**a**) β = 10 C°/min, (**b**) β = 30 ℃/min, (**c**) β = 50 ℃/min.

Theoretical data and experimental results of co-pyrolysis showed that positive synergy occurred between the SM and PET. As seen from Figure 3, weight loss during co-pyrolysis was greater than the theoretical mean values, which are calculated from SM and PET. The apparent activation energy of co-pyrolysis lowered the activation energy compared to PET pyrolysis, suggesting a reduction in energy consumption for pyrolysis [36].

### 3.2. Kinetic Analysis

**Figure 4:**
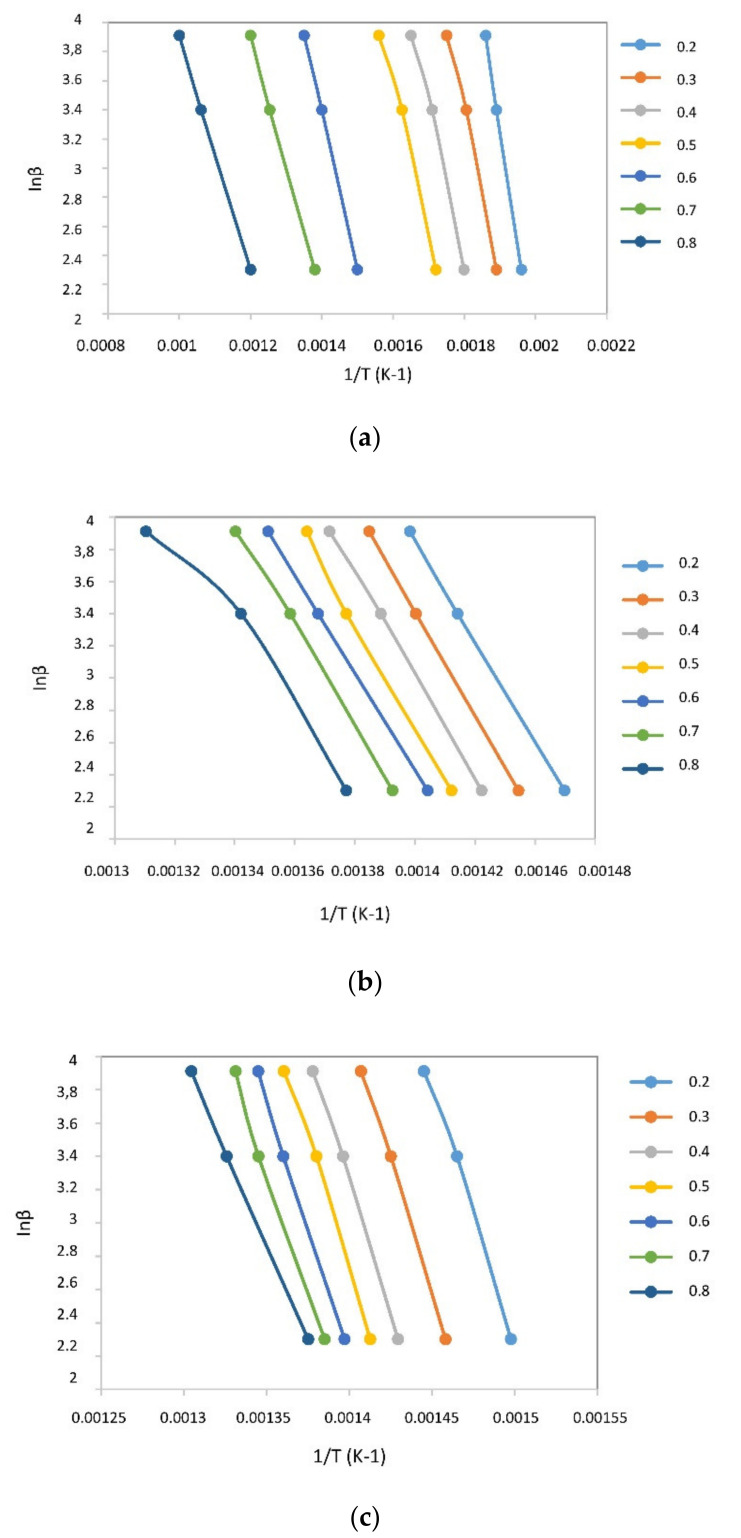
Linear correlation for determining activation energy of SM, PET, and their blend calculated by FWO (**a**), (**b**), (**c**), respectively.

Kinetic study of biomass serves better understanding of reaction mechanisms. Kinetic parameters are used to predict reaction behaviors during thermal degradation of materials [37]. The Ozawa–Flynn–Wall (OFW) model was used for fitting the DTGs of pyrolysis of SM and PET (Figure 4). Activation energy is defined as the minimum amount of energy required to initiate the reaction. Activation energy corresponds to reaction kinetics and reaction mechanisms of pyrolysis. The higher the activation energy, the slower the reaction [38]. The activation energies calculated are shown in Table 3. The correlation coefficient were higher than 0.98, which implied good correlation with experimental data.

The average *E_a_* values of SM, PET and blend estimated with the FWO method were: 86.27 kJ/mol, 241.53 kJ/mol, and 234.51 kJ/mol, respectively. As can be seen from Table 3, activation energies in the conversion degree range of 0.2–0.8, reduced with co-pyrolysis. Reduction activation energy was reported during co-pyrolysis of biomass with plastic waste [36,37,38,39]. Co-pyrolysis resulted in a gradual decrease in average activation energy. This may be due to activation and decomposition of biomass components such as cellulose, hemicellulose, lignin, extractives and other components [40]. Reduction in activation energy depicts easier thermal conversion [41]. The activation energy of PET was found to be greater than SM due to structural differences between biomass and plastic waste [37]. Blending lowered the required activation energy to initiate the reaction.

In calculation of the pre-exponential factors, a 10 °C/min heating rate was used. The pre-exponential factors calculated using the FWO model varied from E+03 s^−1^ to E+16 s^−1^, indicating the occurrence of complex reactions during thermal processing. The pre-exponential factor (*A* ≥ 109) implied a simple complex with high reactivity [41]. The thermodynamic parameters were given in Table 3. The change in enthalpy (Δ*H*) indicated if the reaction process was endothermic or exothermic. Δ*H* values in all conversion degrees were positive, indicating occurrence of endothermic reactions during pyrolysis processes. The difference between activation energy and enthalpy were within the range of 4 kj/mol–6 kj/mol, which implied product formation with a small amount of energy [42]. The results are in agreement with previous studies [43,44].

Entropy change (Δ*S*) determines the reactivity of reaction systems. The negative value of entropy during SM degradation implied that the degree of disorder of the products is much lower than the SM. The higher value of entropy means higher reactivity and shorter reaction times during PET decomposition [45,46]. The blend has shown variations in entropy, and has conducted different behavior than the feedstock. Gibbs free energy (Δ*G*) analysis indicated the amount of energy available from the material. The average values of Δ*G* were found to be 173.36 kJ/mol, 206.09 kJ/mol, and 228.74 kJ/mol for SM, PET, and their blend, respectively. The calculated Δ*G* values have shown that co-pyrolysis of the SM-PET blend has a considerable bioenergy potential.

## 4. Conclusions

In this study, co-pyrolysis of sheep manure and recycled polyethylene terephthalate was examined in order to understand the kinetics and synergetic effects. *TGA* analysis demonstrated the existence of synergistic effects during co-pyrolysis. The apparent activation energies of SM, PET, and their blend were calculated by the FWO method as 86.27, 241.53, and 234.51 kJ/mol, respectively. Higher Gibbs free energy analysis of the blend implied the amount of available green energy from the waste materials. Co-pyrolysis can serve as an alternative waste management method that has significant impact on waste utilization and energy production.

## Figures and Tables

**Table 1:** Proximate and ultimate analyses of samples.

Proximate Analysis (As Received Basis)		
	SM	PET
Moisture, %	19.42	0.65
Volatile Matter, %	38.78	86.12
Fixed Carbon, %	12.88	13.19
Ash, %	28.92	0.04
Ultimate Analysis (Dry Basis)		
C, %	22.50	63.5
H, %	3.48	4.7
N, %	3.09	0.05
S, %	0.50	0.03
O, % (by difference)	32.73	31.68
LHV (kJ/kg)	13.55	20.32

**Table 2:** Characteristic temperatures of pyrolysis.

	SM			PET			Blend		
*β* (°C/min)	10	30	50	10	30	50	10	30	50
*T_i_* (°C)	210.65	217.49	229.72	386.05	403.50	409.86	370.85	390.07	400.15
*T_max_*(°C)	314.94	329.05	336.20	435.70	459.80	465.91	430.50	450.46	470.50
*T_f_* (°C)	486.63	502.36	536.29	469.05	495.86	523.45	489.50	540.96	560.00
Total Weight Loss, %	61.83	60.13	53.45	98.63	93.50	91.32	77.94	74.77	73.56

**Table 3:** Kinetic parameters for the pyrolysis of cattle manure, recycled polyester and their blend.

α	*E_a_* (kJ/mol)	*A* (s^−1^)	*R* ^2^	Δ*H* (kJ/mol)	Δ*G* (kJ/mol)	Δ*S* (J/mol K)
	SM					
0.2	126.64	1.30 × 10^+09^	0.9915	121.75	171.38	−84.39
0.3	91.83	7.63 × 10^+05^	0.9957	86.94	172.95	−146.26
0.4	86.29	2.31 × 10^+05^	0.9932	81.40	173.26	−156.19
0.5	80.37	6.41 × 10^+04^	0.9990	75.48	173.60	−166.86
0.6	85.08	1.78 × 10^+05^	0.9965	80.19	173.32	−158.36
0.7	70.30	7.15 × 10^+03^	0.9955	65.41	174.26	−185.09
0.8	63.39	1.57 × 10^+03^	0.9941	58.50	174.76	−197.70
***Average***	86.27			81.38	173.36	
	**PET**					
0.2	246.50	1.44 × 10^+16^	0.9990	240.61	205.95	48.90
0.3	255.84	7.28 × 10^+16^	1.0000	249.94	205.73	62.37
0.4	251.89	3.67 × 10^+16^	0.9975	246.00	205.82	56.68
0.5	260.83	1.73 × 10^+17^	0.9978	254.94	205.62	69.58
0.6	239.19	4.04 × 10^+15^	1.0000	233.30	206.13	38.34
0.7	244.90	1.09 × 10^+16^	0.9947	239.01	205.99	46.58
0.8	191.53	9.94 × 10^+11^	0.9752	185.64	207.44	−30.75
***Average***	241.53			235.63	206.09	
	**Blend (1:1 wt%)**					
0.2	244.14	2.47 × 10^+16^	0.9980	238.37	201.21	53.57
0.3	250.40	7.50 × 10^+16^	0.9987	244.63	201.07	62.81
0.4	248.42	5.28 × 10^+16^	0.9985	242.66	201.11	59.89
0.5	245.89	3.37 × 10^+16^	0.9977	240.12	201.17	56.15
0.6	241.63	1.58 × 10^+16^	0.9965	235.86	201.27	49.86
0.7	231.66	2.69 × 10^+15^	0.9973	225.89	201.52	35.15
0.8	179.44	2.44 × 10^+11^	0.9901	173.67	202.99	−42.26
***Average***	234.51			228.74	201.48	
